# Attitudes and beliefs about how chefs can promote nutrition and sustainable food systems among students at a US culinary school

**DOI:** 10.1017/S1368980021003578

**Published:** 2022-02

**Authors:** Jaclyn Bertoldo, Robert Hsu, Taylor Reid, Allison Righter, Julia A Wolfson

**Affiliations:** 1 Department of International Health, Johns Hopkins University Bloomberg, School of Public Health, 615 North Wolfe Street, Baltimore, MD 21205, USA; 2 Department of Health Management and Policy, University of Michigan, School of Public Health, Ann Arbor, MI, USA; 3 School of Liberal Arts and Food Studies, The Culinary Institute of America, Hyde Park, NY, USA; 4 School of Culinary Science and Nutrition, The Culinary Institute of America, Hyde Park, NY, USA

**Keywords:** Culinary students, Chefs, Nutrition, Sustainability, Public health, Food priorities, Attitudes

## Abstract

**Objective:**

Chefs have the potential to influence diet quality and food systems sustainability through their work. We aimed to assess the attitudes and perceptions of culinary students about nutrition and sustainability as part of their roles, responsibilities and future work as chefs.

**Design:**

We surveyed students attending the Culinary Institute of America (CIA) in the fall of 2019 (*n* 546). Descriptive statistics compared food priority rankings and Likert-scale distributions of nutrition and sustainability attitudes and beliefs. Adjusted generalised linear models were used to evaluate whether there were differences in attitudes and beliefs across demographic groups.

**Setting:**

The CIA, a private, not-for-profit college and culinary school with US campuses in New York, California and Texas.

**Participants:**

Students >18 years old currently enrolled in any of the school’s associate’s or bachelor’s degree programs.

**Results:**

Students agreed that chefs should be knowledgeable about nutrition (96·0 %) and the environmental impact of their ingredients (90·8 %) but fewer considered healthfulness (57·8 %) and environmental impact (60·2 %) of their food to be primary considerations in their career as a chef. Taste was the primary factor influencing culinary students’ food choices but food priorities differed by race/ethnicity.

**Conclusions:**

Culinary students believe nutrition and sustainability are important. Opportunities exist to empower them with knowledge and skills for promoting public health and sustainable food systems in their future work as chefs.

There is an urgent need to promote adoption of healthier diets and sustainable food systems in order to reduce the burden of lifestyle-related morbidity and mortality and preserve the world’s ecosystems for future generations^(1)^. Chefs have emerged as key stakeholders in efforts to positively shape consumer eating habits and food systems transformation^(2,3)^. Food away from home now makes up close to 1/3 of total calories consumed and more than 1/2 of household food expenditures^(4)^, making chefs uniquely positioned to influence dietary intake of Americans via the nutritional quality of food away from home ^(5–7)^. In addition, chefs can promote sustainable food systems and mitigate food systems-related climate change by adapting their menus and business practices^(5,8–10)^. The potential for impact extends beyond more traditional full service restaurants to encompass other settings and sectors that reach large numbers of people including institutional food service (e.g. K-12 education, universities and hospitals), chain restaurants and food and beverage companies^(11)^.

The rise of the ‘celebrity chef’ has popularised chefs as food experts and sources of nutrition information, expanding the chef’s influence outside of the kitchen to help shape public attitudes, corporate interests and government policy on nutrition and sustainability issues^(12–15)^. Previous work has demonstrated that chefs tend to have positive attitudes towards nutrition and their role in influencing food choices however, this does not necessarily translate into increased nutritional knowledge or more healthful food preparation^(16–19)^. While chefs are increasingly implementing sustainable menu and business practices, they consistently rank sustainability factors such as local sourcing, environmental/humane animal treatment and organic foods below other priorities such as taste, convenience, nutrition and cost^(8,15)^.

Despite recognising the opportunity, they have to enhance population and environmental health, chefs have not yet broadly embraced their roles as agents of change in the food system. Many culinary schools are now incorporating nutrition and sustainability into their curriculums in acknowledgement of the need to prepare chefs for expanded roles and responsibilities in these areas^(20–23)^. For instance, the American Culinary Federation Education Foundation, Inc. Accrediting Commission requires a minimum of 30 h of theoretical and applied learning in nutrition for over 200 of its accredited postsecondary culinary programs^(24)^. In 2016, The Ministry of Training, Colleges and Universities mandated that culinary schools in Ontario incorporate sustainability into their curriculum requirements^(25)^, and several US culinary schools now offer certificates in sustainable food systems or integrate sustainability concepts into their degree programs^(26–29)^.

A small number of studies have explored attitudes of culinary students about nutrition^(17,21,30)^, but the role of sustainability in culinary education and attitudes towards sustainability for chefs in training has not been studied. As calls strengthen to incorporate sustainability into dietary guidance and practice^(31,32)^, more must be done to understand how the next generation of chefs view healthy eating and sustainable practices as part of their future roles, and how nutrition and sustainability are balanced against other prominent factors affecting food choices such as taste, price and convenience. The purpose of this study was to examine food choice priorities of undergraduate students enrolled in a degree program at a US culinary school (‘culinary students’) and to assess how they perceive nutrition and sustainability as part of their roles, responsibilities and future work as chefs.

## Methods

We designed a web-based survey using the Survey Gizmo platform to assess factors influencing personal food choices, attitudes and beliefs about chefs’ roles in the food system, and intention to incorporate nutrition and sustainability into a career as a chef among students attending The Culinary Institute of America (CIA). Survey questions were informed by previous literature on food choice priorities^(33)^ and attitudes and beliefs of chefs^(8,16–18,21)^, and were reviewed by experts for face validity prior to initiating the survey. The CIA is a private, not-for-profit college with campuses in New York, California, Texas and Singapore, offering associate degrees, bachelor’s degrees, master’s degrees and certificates in a range of culinary and food business specialties. The total undergraduate enrolment at the CIA in 2019 was 3668 students.

The survey was distributed via email to all CIA students >18 years old currently enrolled in any associate or bachelor’s degree program across their three US campuses. Singapore students, graduate students and any student <18 years old at the time the email list was pulled were excluded. A total of 2609 students were sent an email inviting them to participate in the survey, and data were collected between 19 September 2019 and 5 October 2019 with three email reminders sent throughout the data collection period. Participation in the survey was voluntary, and participants were required to provide consent before answering the survey questions. A total of 551 participants completed the survey, representing a 21 % completion rate. Participants were incentivised with the option of being entered into a raffle for one of sixteen $25 gift cards after completing the survey. Respondents who completed the survey but who did not consent to participate in the study (*n* 5) were excluded from the analysis, creating a final analytic sample of 546 CIA students. All data received by the researchers was de-identified, and this study was determined to be exempt (category #2) by the University of Michigan Institutional Review Board.

### Measures

#### Factors influencing food choices (at home or when eating out at a restaurant)

Respondents were asked to rank the following seven factors in order of importance (1 = most important, 7 = least important) when deciding what to eat for a meal, either at home or in a restaurant: taste, price, convenience, sustainability, healthfulness, seasonality and locally grown. Taste, price and convenience were chosen based on primary drivers of food choices previously documented in the general US population^(33)^.

#### Attitudes and beliefs about nutrition and sustainability

Attitudes and beliefs were assessed by asking respondents to reflect on a series of statements relating to nutrition and sustainability, indicating the level to which they agreed with each statement. All items were measured on a five-point Likert scale from strongly disagree (1), disagree (2), neutral (3), agree (4) and strongly agree (5). Prompts related to general attitudes included the statements ‘Poor diet quality is a big problem in the US,’ and ‘Food system contributions to climate change are a big problem.’ Respondents also reflected on their beliefs about the importance of chefs having nutrition and sustainability knowledge (e.g. ‘It is important for chefs to learn about nutrition,’ and ‘It is important for chefs to understand the environmental impact of their ingredients’), and whether chefs have a responsibility to help address obesity and other diet-related health problems or climate change through their menu decisions.

#### Personal goals about incorporating nutrition and sustainability into career

As the survey participants are students training to become chefs, we also evaluated intentions for incorporating nutrition and sustainability into their future careers. Using a five-point Likert scale from strongly disagree (1), disagree (2), neutral (3), agree (4) and strongly agree (5), respondents were asked to rank their level of agreement with statements such as ‘I hope to promote healthy eating habits through my work as a chef’ and ‘I hope to promote environmentally sustainable local food systems through my work as a chef.’

#### Open-ended survey questions

Survey respondents were asked open-ended questions inviting them to share their opinions on whether chefs are appropriate role models for healthy eating and reducing food system contributions to climate change (why or why not?), and to provide suggestions for how chefs might promote healthy and environmentally sustainable foods and behaviours through their work.

#### Demographic measures

Covariates included gender, age (18–19 years, 20–22 years, ≥23 years), race/ethnicity (Non-Hispanic White, Non-Hispanic Black, Hispanic, other), year in program (1st, 2nd, 3rd, 4th) and Pell Grant status. Pell Grants are awarded by the federal government to low-income students who demonstrate exceptional financial need and receipt of a Pell Grant is used as a proxy for socioeconomic status in this sample.

### Analysis

Analyses were conducted using Stata, Version 16.1 (StataCorp., LP), with significance set at *P* < 0·05. Descriptive statistics for each measure (frequencies, percentages, means and confidence intervals) were generated for the full survey sample and across the demographic covariates (gender, age category, race/ethnicity, year in program and Pell grant status). Stacked graphs were created to illustrate the proportions of respondents ranking each of the seven factors influencing food choices in order of importance and to describe the unadjusted Likert-scale distributions showing levels of agreeance across the different nutrition- and sustainability-themed statements.

We ran two sample *t*-tests to explore significant differences in attitudes and beliefs for gender and Pell grant status, and ANOVA with Tukey post-hoc tests for race/ethnicity, age category and year in program. Likert scale responses were re-coded into dichotomous variables, with 1 indicating agreement with the statement (agree (4) or strongly agree (5)) and 0 indicating either a neutral response (3) or disagreement (disagree (2), strongly disagree (1)). Due to the fact that affirming the attitudes and beliefs about nutrition and sustainability was a relatively common outcome, we chose to use generalised linear models with a Poisson family, log link and robust standard errors to calculate the relative risk (RR) of agreeing with each statement of attitudes and beliefs across gender, race/ethnicity, age, class year and Pell grant status. These methods are described in more detail elsewhere^(34)^. Separate, fully adjusted models were estimated for each measure of attitudes and beliefs about nutrition and sustainability.

Qualitative analyses used a thematic analysis approach^(35)^. Open-ended survey answers were given initial codes by two members of the research team using an inductive and iterative process. Initial codes were defined using a line by line reading of the data and were then grouped into broader themes. Coding was done ‘by hand’ in Microsoft Excel and Word; a qualitative data analysis software was not used to facilitate coding.

## Results

Table 1 describes the demographic characteristics of the sample and total student population. Students who responded to the survey were more likely to be female (60·3 %) compared to 47·6 % female among the overall student population. Race/ethnicity distribution of the sample more closely mirrored that of all students with 52·8 % identifying as non-Hispanic white (43·6 % in the total student population), 6·2 % as non-Hispanic Black (7·2 % in the total student population) and 13·4 % as Hispanic (15·7 % in the total student population). The majority (51·3 %) of the respondents were 18–19 years old and were in the first year of their associate or bachelor’s program (56·6 %). The CIA has a large proportion of students (54 %) pursuing a 2-year associate’s degree which may explain why such a high percentage of survey respondents were in their first or second year. Thirty-three percent of the respondents were Pell Grant recipients which is slightly higher than the proportion of Pell Grant recipients among all students (28·8 %).

**Table 1:** Demographic characteristics of the study sample and total student population

Study sample	*n*	Percent	All students	*n*	Percent
Total	546	100	Total	3668	100
Sex			Sex		
Female	329	60·3	Female	1730	47·6
Male	204	37·4	Male	1863	50·8
Not reported	13	2·4	Not reported	75	2·0
Race/ethnicity			Race/ethnicity		
Non-Hispanic White	288	52·8	Non-Hispanic White	1598	43·6
Non-Hispanic Black	34	6·2	Non-Hispanic Black	265	7·2
Hispanic	73	13·4	Hispanic	574	15·7
Other	116	21·3	Other/not reported	1231	33·6
Not reported	35	6·4			
Age			Age		
18–19 years	280	51·3	18–24 years	2772	75·6
20–22 years	140	25·6	25–64 years	886	24·2
≥23 years	126	23·1			
Year in program			Year in program*		
1st year	309	56·6	1st year	–	–
2nd year	147	26·9	2nd year	–	–
3rd year	55	10·1	3rd year	–	–
4th year	35	6·4	4th year	–	–
Pell grant status			Pell grant status		
No	367	67·2	No	2661	71·2
Yes	179	32·8	Yes	1057	28·8

* Data not available.

### Factors influencing food choices (at home or when eating out at a restaurant)

**Fig. 1:**
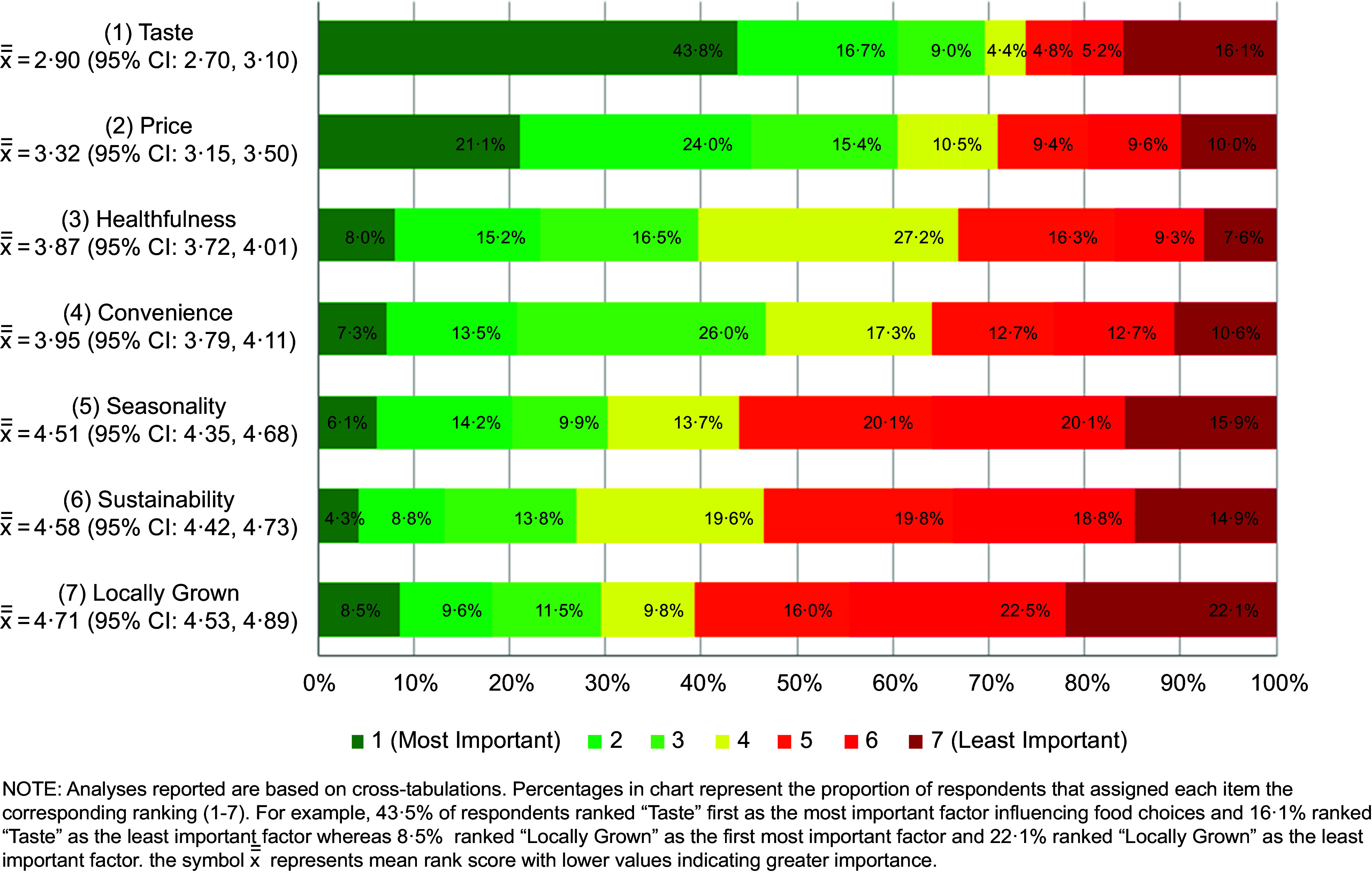
Rank order of influences on food choices (at home or when eating out in a restaurant)

Overall, taste was ranked as the most important factor influencing culinary students’ food choices with 43·8 % of respondents ranking it as their top priority (mean rank score = 2·90 (95 % CI 2·70, 3·10)). Taste was followed by price (3·32 (95 % CI 3·15, 3·50)), healthfulness (3·87 (95 % CI 3·72, 4·01)), convenience (3·95 (95 % CI 3·79, 4·11)), seasonality (4·51 (95 % CI 4·35, 4·68)), sustainability (4·58 (95 % CI 4·42, 4·73)) and locally grown (4·71 (95 % CI 4·53, 4·89)) (Fig. 1). The rank order of taste, price, healthfulness and convenience differed by race/ethnicity, with non-Hispanic Black respondents ranking price as the top priority (2·73 (95 % CI 2·03, 3·44)) followed by taste (3·13 (95 % CI 2·27, 3·98)), convenience (3·69 (95 % CI 3·07, 4·31)) and healthfulness (3·77 (95 % CI 3·22, 4·31)), and Hispanic participants ranking convenience (3·90 (95 % CI 3·45, 4·34)) as the third most important factor after taste (2·84 (95 % CI 2·31, 3·38)) and price (3·09 (95 % CI 2·59, 3·59)), and ahead of healthfulness (3·96 (95 % CI 3·56, 4·35)) (see online Supplemental Fig. 1). Respondents in the youngest age category (18–19 years old) also ranked convenience (3·83 (95 % CI 3·61, 4·04)) behind taste (2·88 (95 % CI 2·60, 3·16)) and price (3·26 (95 % CI 3·02, 3·51)) but ahead of healthfulness (3·95 (95 % CI 3·74, 4·15)) while the oldest age category (≥23 years) ranked healthfulness (3·66 (95 % CI 3·35, 3·98)) as the second most important factor after taste (2·64 (95 % CI 2·25, 3·04)), followed by price (3·72 (95 % CI 3·34, 4·10)) then convenience (4·10 (95 % CI 3·76, 4·43)). The three environmental factors (seasonality, locally grown, sustainability) always ranked below the other factors, a trend that was consistent across gender, age, class year, race/ethnicity and Pell grant status.

### Attitudes and beliefs among culinary students about nutrition and sustainability

**Fig. 2:**
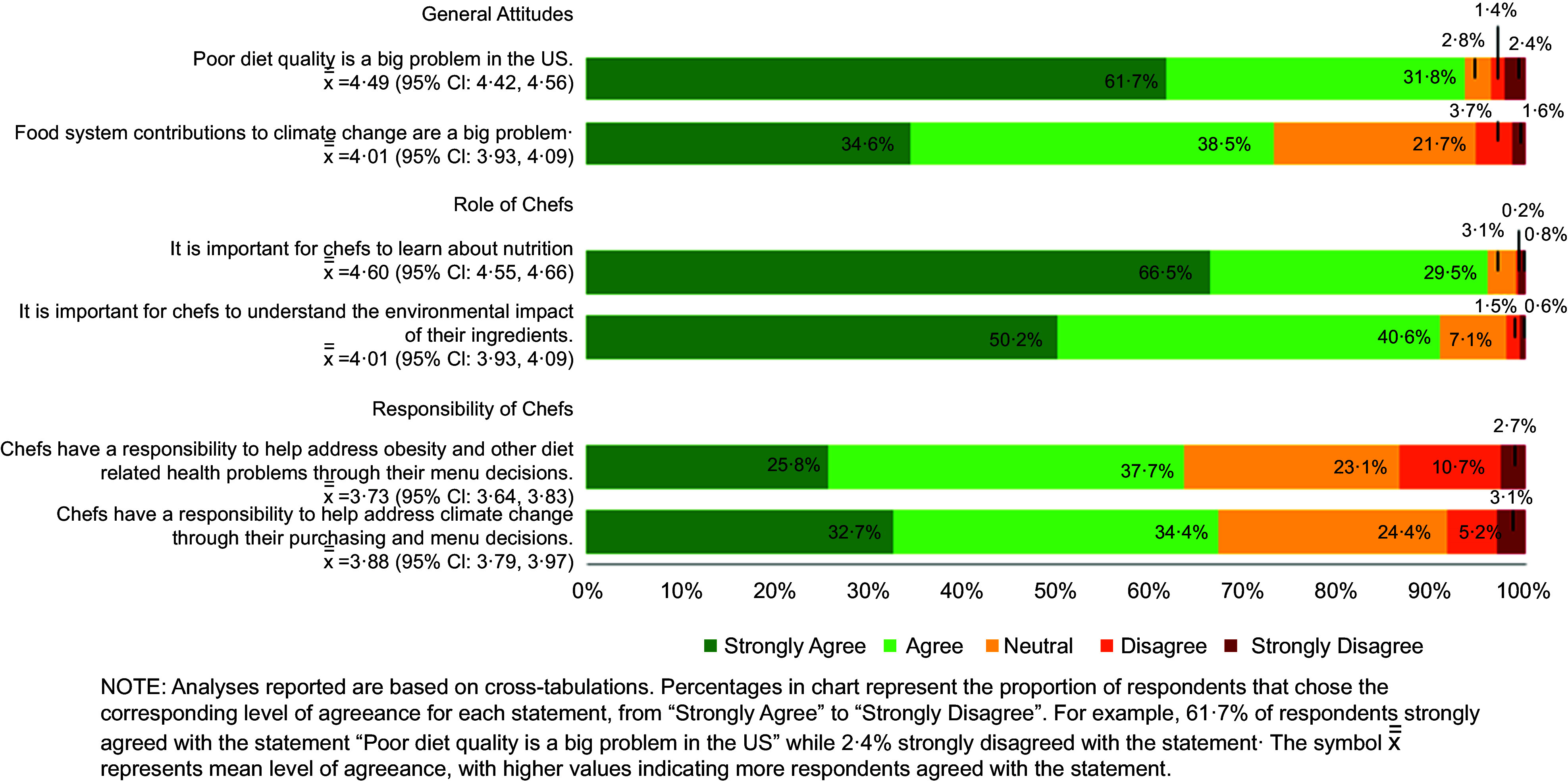
Unadjusted distribution of culinary student attitudes and beliefs about nutrition and sustainability

Most culinary students surveyed agreed or strongly agreed that it is important for chefs to learn about nutrition (96·0 %) and the environmental impact of their ingredients (90·8 %) (Fig. 2). More respondents agreed that poor diet quality is a big problem in the USA (93·5 %) compared to food system contributions to climate change (73·1 %), yet slightly more respondents believe it is part of the chef’s role to help address climate change through menu and purchasing decisions (67·1 % agree or strongly agree) than believe chefs have a responsibility to help address obesity and other related health problems through their menu decisions (63·5 % agree or strongly agree).

Fully adjusted models revealed that females were more likely to agree that diet quality is a big problem in the USA (RR 1·06 (95 % CI 1·00, 1·12), *P* = 0·04) but this did not translate into any significant gender differences in beliefs about nutrition and sustainability as part of a chef’s roles and responsibilities.

Non-Hispanic Black students were significantly less likely to agree that chefs have a responsibility to address climate change through their purchasing and menu decisions (RR 0·57 (95 % CI 0·37, 0·88), *P* = 0·01) compared to other racial/ethnic groups (Table 2). Students in their 4th academic year were significantly more likely to agree that food systems contributions to climate change are a big problem (RR 1·29 (95 % CI 1·07, 1·55), *P* = 0·006), that it is important for chefs to understand the environmental impact of their ingredients (RR 1·12 (95 % CI 1·05, 1·20), *P* < 0·001) and that chefs have a responsibility to address climate change through their purchasing and menu decisions (RR 0·57 (95 % CI 0·37, 0·88, *P* = 0·02).

**Table 2:** Adjusted model of culinary student attitudes and beliefs about nutrition and sustainability

	General attitudes				Role of chefs				Responsibility of chef			
	Poor diet quality is a big problem in the USA		Food system contributions to climate change are a big problem		It is important for chefs to learn about nutrition		It is important for chefs to understand the environmental impact of their ingredients		Chefs have a responsibility to help address obesity and other diet-related health problems through their menu decisions		Chefs have a responsibility to help address climate change throughout their purchasing and menu decisions	
	RR	95 % CI	RR	95 % CI	RR	95 % CI	RR	95 % CI	RR	95 % CI	RR	95 % CI
Sex												
Male	Reference		Reference		Reference		Reference		Reference		Reference	
Female	1·06*	1·00, 1·12	0·92	0·83, 1·03	1·00	0·96, 1·04	1·07	0·95, 1·08	1·11	0·96, 1·29	0·96	0·84, 1·09
Race/ethnicity												
Non-Hispanic White	Reference		Reference		Reference		Reference		Reference		Reference	
Non-Hispanic Black	0·92	0·79, 1·06	0·75	0·54, 1·04	1·00	0·93, 1·08	0·90	0·76, 1·07	0·86	0·61, 1·20	0·57*	0·37, 0·88
Hispanic	0·96	0·88, 1·05	0·93	0·78, 1·12	0·97	0·90, 1·05	1·00	0·92, 1·10	1·02	0·82, 1·26	1·03	0·85, 1·24
Other	0·96	0·89, 1·02	1·16**	1·04, 1·31	1·00	0·95, 1·04	0·96	0·89, 1·05	1·05	0·88, 1·26	0·90	0·75, 1·07
Age												
18–19 years	Reference		Reference		Reference		Reference		Reference		Reference	
20–22 years	0·98	0·90, 1·06	1·04	0·89, 1·21	1·01	0·96, 1·05	1·01	0·94, 1·10	1·01	0·83, 1·23	1·15	0·96, 1·36
≥23 years	0·98	0·92, 1·05	1·03	0·90, 1·19	1·00	0·96, 1·05	1·00	0·92, 1·08	0·96	0·80, 1·15	1·06	0·89, 1·25
Year in program												
1st year	Reference		Reference		Reference		Reference		Reference		Reference	
2nd year	0·99	0·93, 1·05	1·09	0·96, 1·24	1·01	0·96, 1·05	1·04	0·97, 1·11	1·16	0·99, 1·35	1·17*	1·01, 1·36
3rd year	1·00	0·91, 1·10	1·04	0·85, 1·28	0·97	0·90, 1·05	1·01	0·90, 1·12	0·86	0·63, 1·17	0·96	0·74, 1·24
4th year	1·07	0·98, 1·16	1·29**	1·07, 1·55	1·00	0·93, 1·08	1·12***	1·05, 1·20	1·24	0·95, 1·63	1·29*	1·03, 1·60
Pell grant status												
No	Reference		Reference		Reference		Reference		Reference		Reference	
Yes	0·96	0·91, 1·02	0·97	0·85, 1·09	1·00	0·95, 1·04	0·98	0·91, 1·05	0·96	0·82, 1·12	0·91	0·78, 1·05

Generalised linear models with a Poisson family, log link and robust error variance comparing the outcome of agree or strongly agree (1) to neutral, disagree, strongly disagree (0).

All models were adjusted for sex, race/ethnicity, age, year in program and Pell grant status.

* *P* < 0 05.

** *P* < 0 01.

*** *P* < 0 001.

### Culinary student goals about incorporating nutrition and sustainability into career as chef

**Fig. 3:**
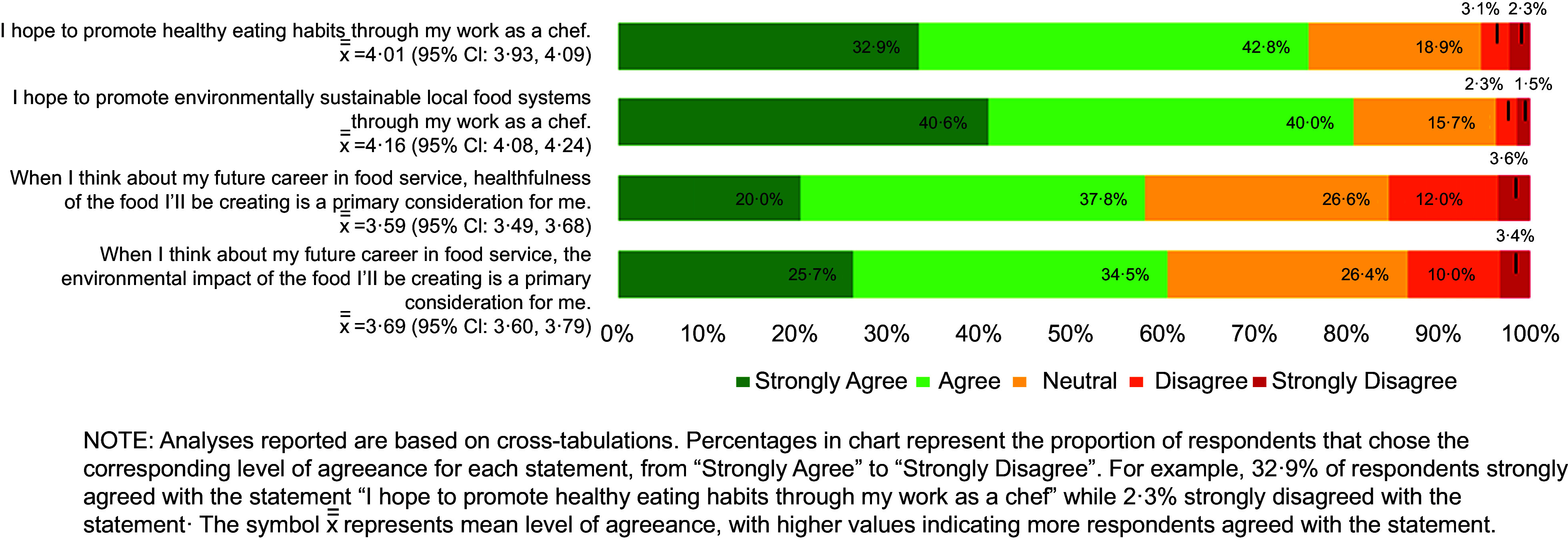
Culinary student goals about incorporating nutrition and sustainability into a career as chef

Most culinary students surveyed said that they plan to promote healthy eating habits and environmentally sustainable food systems through their work as chefs (75·7 % and 80·6 % agree/strongly agree, respectively). However, fewer students consider the health and environmental impact of the food they will create to be a primary consideration in their future careers (57·8 % and 60·2 % agree/strongly agree, respectively) (Fig. 3).

### Open responses on whether the public should look up to chefs as role models for healthy eating and reducing food system contributions to climate change

Table 3 describes key themes and excerpts from the open response questions. Seven primary themes under healthy eating were identified from the data: (1) Knowledge; (2) Individual Choice; (3) Voice and Influence; (4) Specialisation; (5) Not Chef’s Job; (6) Good Food Not Healthy and (7) Improve Taste. Themes related to food systems contributions to climate change included: (1) Someone Else; (2) Sourcing; (3) Voice and Influence; (4) Food Waste; (5) Agriculture; (6) Contributors and (7) Knowledge.

**Table 3:** Open response themes and key quotations on whether the public should look up to chefs as role models for healthy eating and reducing food system contributions to climate change

Healthy eating (*n* 342)		Food systems contributions to climate change (*n* 328)	
Knowledge—Chefs have more food/nutrition knowledge	‘Chefs are trained professionals in the culinary world, making them more knowledgeable than most about nutrition.’ - First year student in Culinary Science ‘I do believe it’s appropriate because chefs are the people who should be the most knowledgeable about food, and thus should be the most knowledgeable on food’s health related connections.’ - First year student in Culinary Science “Yes, we are the field experts on what we are cooking we should therefore be role models, to the public.” - First year student in Culinary Arts	Someone Else—Climate change action is not just a chef’s responsibility	‘No. Chefs alone do not just contribute to climate change. The factories that some of the major foods come out of do. Look at the companies, not the chefs.’ - First year student in Food Business Management ‘People should not only look to chefs, they should look to anyone looking to reduce food system contributions to climate change.’” - First year student in Culinary Science
Individual Choice—Health is an individual choice	‘No. I believe it is up to the individual to understand their own bodies and take care of themselves. There are thousands upon thousands of dietary restriction. We cannot accommodate for everyone.’ - First year student in Culinary Arts ‘No, it is a persons responsibility to be healthy for themselves and select healthy options from the menu if a chef decides to provide them.’ - First year student in Culinary Arts ‘People are responsible for their own actions and decisions. It is irresponsible to solely depend on a chef or expect that a chef is always going to serve the healthiest option.’ - First year student in Culinary Arts	Sourcing—How chefs buy ingredients is important in addressing climate change	‘Yes. Climate change is a huge issue and chefs can easily reduce what they add to it by choosing better ingredients.’ - First year student in Baking & Pastry Arts ‘Yes. As some of the main food purchasers we should be the ones looked at first on reducing food system concerns for climate change.’ - Second year student in Baking & Pastry Arts
Voice and Influence—Chefs have a platform and can influence others	‘I believe chefs should be looked at as the ones leading the conversation for what healthy cooking and eating looks like.’ - Fourth year student in Applied Food Studies ‘I believe that the public should be able to look up to chefs as role models for healthy eating and cooking because if professionals are doing something, it will influence others to also do it.’ - First year student in Food Business Management ‘Chefs should be the societal figure for healthy eating and change for the future because the ingredients and the dishes a chef makes has an impact and has a wide audience.’ - First year student in Food Business Management	Voice and Influence—Chefs have a platform and can influence others	‘Yes, because, again, we are the cultivators of the food eaten by the masses. We also can control how the system affects the world around us. We should be making a constant effort to recognize the effects of our industry on climate change.’ - First year student in Food Business Management ‘Yes, people are easily influenced by trends in the industry. If they notice more changes and awareness in restaurants, then they will want to change their ways for the sake of future generations.’ - First year student in Baking & Pastry Arts
Specialisation—Some chefs may specialise in healthy food but not all chefs	‘Some might chose that as their focus, but it should not be expected of chefs to be healthy.’ - Third year student in Applied Food Studies ‘It depends on what type of chef it is because some chefs don’t focus on healthy cooking and some chefs do.’ - First year student in Culinary Arts	Food Waste—Chefs have a responsibility to reduce food waste	‘Yes! Looking up to chefs on how they deal with food waste & how to run a low food waste kitchen could lead to a drastic change in how Americans cook.’ - Second year student in Culinary Arts ‘I think it is appropriate as long as they are doing what is in their power to contribute as little waste in product and energy as possible.’ - Second year student in Applied Food Studies
Not Chef’s Job—It’s not chefs’ responsibility to promote healthy eating or make healthy food	‘No. Chefs primary concern is to make the food their customers want to eat.’ - Fourth year student in Culinary Arts ‘No, it isn’t a chef’s job to be a role model for healthy eating, their job is to put out good food.’ - First year student in Culinary Arts	Agriculture—Connection between chefs and agriculture	‘Absolutely, as climate change becomes the primary concern of the next few decades, it is the chefs duty to do all they can in supporting ethical and sustainable agricultural practices.’ - First year student in Applied Food Studies
Good Food Not Healthy—Chefs make food that tastes good, not healthy	‘No, because chefs aren’t only looking for healthiness. They’re looking for great taste which often leads to using ingredients that taste good but is not good for you.’ - First year student in Hospitality Management ‘No, because chefs make what taste good not necessarily what is the healthiest so they aren’t good role models.’ - Second year student in Baking & Pastry Arts ‘Sometimes. Often chefs are more concerned with food tasting good than being good for you.’ - Second year student in Culinary Science	Contributors—Chefs activities and choices contribute to climate change	‘Yes because what we use in cooking can have negative effects on climate change for example fish and the over fishing of it.’ - Second year student in Hospitality Management
Improve Taste—Chefs have skills to make healthy food taste good	‘Yes because people think that chefs would be the people to make the healthy food taste good compared to just steaming or cooking the healthy food without anything making it interesting and tasty.’ - First year student in Baking & Pastry Arts ‘I think it’s appropriate because Chefs can be creative and make something unhealthy into something that is better for you with the same taste.’ - First year student in Food Business Management	Knowledge—Chefs have more knowledge about food and environment than others	‘Yes because chefs know more about food, where it comes from and how to best use it.’ - First year student in Baking & Pastry Arts

Most participants reflected positively on the public looking to chefs as role models for healthy eating and reducing food system contributions to climate change. Many culinary students described chefs as food experts with unique knowledge that can be used to promote healthy eating. For example, one first year student in the Culinary Science program noted, ‘*Chefs are trained professionals in the culinary world, making them more knowledgeable than most about nutrition.*’ Perspectives on the chef’s role in reducing food system contributions to climate change were more closely tied to implementing sustainable practices, such as choosing what to purchase and where to purchase it from. A second-year student studying Baking and Pastry Arts explained, *‘As some of the main food purchasers we should be the ones looked at first on reducing food system concerns for climate change.’*

A theme that emerged across both nutrition and environmental perspectives is the belief that chefs have a platform that should be utilised to positively influence others to adopt more healthful and sustainable eating behaviours. *‘I believe chefs should be looked at as the ones leading the conversation for what healthy cooking and eating looks like,’* stated a fourth-year student in Applied Food Studies. A first-year student in the Baking and Pastry Arts added, *‘Yes, people are easily influenced by trends in the industry. If they notice more changes and awareness in restaurants, then they will want to change their ways for the sake of future generations.’*

The open-ended responses also revealed why some participants do not see chefs as role models for healthy eating and sustainability, primarily the belief that this should not be the chef’s responsibility. According to a first-year student in the Culinary Arts program, *‘People are responsible for their own actions and decisions. It is irresponsible to solely depend on a chef or expect that a chef is always going to serve the healthiest option.’* Another first-year student in the Food Business Management program expressed, *‘No. Chefs alone do not just contribute to climate change. The factories that some of the major foods come out of do. Look at the companies, not the chefs.’*

Some respondents described how factors guiding food choices can be at odds with one another, for instance, that healthfulness and sustainability of a food can negatively impact taste and price. *‘No, because chefs need to make money and what sells is delicious food. Healthier cooking is more challenging to make it taste good and through all the work we have to do as cooks, it is low on my priority list,’* remarked a second-year student studying Culinary Arts. A second-year student in the Food Business Management program explained, *‘Some chefs may be proud of their sustainable menus, but many chefs tend to prioritize cost and flavor over sustainability in ingredients.’*

*Open responses on what chefs can do to help promote healthy eating and environmentally sustainable eating habits among the public.*

The students surveyed offered many suggestions for how chefs can promote healthy eating and environmental sustainability through their work. The responses can be categorised into three primary opportunity areas for chefs to make a positive impact:

1. Through menu and purchasing decisions

‘The most important thing chefs can do is, if they are able to and willing, to revamp their menus to include more plant-based protein items while reducing the amount of meat selection or size of said selections. Or adding to the menu, more healthful items that are intriguing to the public and not just salad.’ – Fourth-year student in Culinary Science program

‘Buying local, chefs should always support local farms and reduce their food miles. They should also practice seasonality and plan their menus around what is in season.’ – Second-year student in Food Business Management program

1. Through acting as educators and influencers

‘Lifelong learning and educating oneself. Our changing world relates to our food and its availability. Chefs can provide quality, healthy food. Provide demos and food education in our schools and colleges. Provide healthy options as opposed to fast food or microwavable. Educate the public.’ – Second-year student in Culinary Arts program

‘Speak to each guest and write on your menu and social media about your sustainability practices and their impact.’ – Second-year student in Culinary Science program

1. Through using culinary skills to make healthy and sustainable options more appealing and accessible to the public

‘The most important thing that chefs can do to promote healthy eating habits is providing solutions to the cliché problem of unappetising healthy food. Show that it tastes good and people will want to eat more of it.’ – Third-year student in Applied Food Studies program

‘Chefs should become educated on how to produce healthy food that tastes delicious on the lowest possible budget. They should use nutritional ingredients and cooking methods that turn cheap ingredients into healthy meals and somehow disseminate that knowledge to the public. Unhealthy food is often popular because of how fast, cheap and convenient it is to obtain. It is vital to create healthy food that can compete with those advantages.’ – First-year student in Culinary Science program.

## Discussion

This study explored the food priorities and attitudes of students at a US culinary school, assessing how they view nutrition and sustainability as part of their future role as stakeholders in the food system. To our knowledge, this is the first study to explore attitudes about both nutrition and sustainability among culinary students. Findings indicate that these future culinary professionals generally believe nutrition and sustainability are important and support these issues as part of chefs’ roles and responsibilities. However, there was variability in how and to what extent these students believe they should apply nutrition and sustainability in their careers. Similar to research in other populations^(8,36)^, taste was the primary driver of personal food decisions amongst this group of culinary students. Other important factors were price, healthfulness and convenience, however the order of these priorities differed by race/ethnicity and between the youngest and oldest age groups.

While there was agreement that it’s important for chefs to be knowledgeable about nutrition, only slightly more than half of students agreed that healthfulness of food would be a primary consideration in their career. These results are similar to findings from Gillis *et al.* (2020)^(21)^ where 99 % of students at a Canadian chef school agreed that chefs need to know how to prepare nutritious food while 60 % ranked nutrition as ‘very important’ for their future work. Unlike Gillis *et al.*, we did not find gender differences in how culinary students value nutrition as part of a chef’s education or work, even though female students in our study were more likely to agree that dietary quality is a big problem in the USA. Differences in culinary student food priorities had not previously been explored across race/ethnicity. In this sample of culinary students, taste, price and convenience were consistently ranked the highest priorities when it comes to personal food choices, a trend that mirrors the preferences of the general population^(33)^. Healthfulness was a lower priority among non-Hispanic Black and Hispanic students when making personal food decisions, with non-Hispanic Black students ranking price above all other factors and both non-Hispanic Black and Hispanic students ranking convenience ahead of healthfulness. It’s unclear how the different food priorities among these groups might translate into the students’ professional choices as chefs, but future studies should explore the implications of these differences and their potential to contribute to nutrition and health disparities. This reinforces the need to leverage the unique knowledge and growing influence of chefs to source, prepare and promote healthy and sustainable foods that are also tasty, affordable and convenient in order to enhance their appeal across all demographic groups. A small but growing number of chefs and restaurants have embraced this approach, such as a former head chef of the top rated restaurant in the world who now produces school meals in London and the Bronx that cost less than $1·25 each^(37)^, a San Francisco Bay Area startup that provides nourishing and culturally relevant reimbursable meals to school districts across the USA^(38)^, or a fast-casual restaurant chain in Los Angeles that serves healthy and convenient meals at reduced prices in low-income neighborhoods^(39)^.

This is the first study we are aware of that explores attitudes towards food systems sustainability among culinary students. Results revealed that many culinary students believe it is important for chefs to understand the environmental impact of their ingredients and to help address sustainability through their work. Despite participants ranking all environmental factors below healthfulness when making food decisions, more students said they hope to promote sustainable food systems and to make the environmental impact of their food a primary consideration when they become chefs than to encourage healthy eating habits or to consider healthfulness of food prominently in their career. There were differences in attitudes towards environmental concerns across race/ethnicity, with non-Hispanic Black participants being significantly less likely to perceive climate change as part of a chef’s responsibilities to address through their work. Attitudes towards climate change are often more closely associated with political affiliation than race/ethnicity, but previous research has suggested that minorities may be less likely to believe there is scientific consensus on climate change or identify as environmentalists^(40)^. While this association is not well understood, it is notable in this context as culinary students who do not see it as their responsibility to address climate change may be less likely to adopt environmental friendly behaviours when they become chefs. Students in their fourth academic year were more likely to believe that chefs should learn about sustainability and take action to address climate change in their work. This may reflect that students in bachelor degree programs at the CIA have more opportunities to incorporate coursework on sustainable food systems compared to those completing associate’s degrees, or perhaps that students who are interested in sustainability are pursuing more advanced culinary degrees.

Our results suggest that the majority of culinary students surveyed could be willing allies in public health efforts to improve diets and reduce food systems contributions to climate change. This is an encouraging insight and demonstrates the potential to engage chefs at all levels in promoting healthy and sustainable diets. There are several current ‘celebrity chefs’ who have taken on prominent roles in efforts to improve dietary quality and nutrition^(41–43)^. Others have focussed on sustainability and climate change by supporting local food systems^(44)^ and eliminating meat to reduce the climate impact of their menus^(45)^. Aside from ‘celebrity chefs’ and the fine-dining sector, sustainable and healthy menu options are both growing trends in the restaurant industry as a whole, including full service, fast casual and fast food restaurants^(46–48)^. In other areas of the food service sector, schools, hospitals and workplace cafeterias are implementing sustainable practices and responding to demand from customers for healthy options^(11,49–52)^. Sustainable procurement policies are growing in prominence in food service, and chefs who value sustainability are well positioned to make progress in these areas^(53)^. Additionally, a number of strategic initiatives and collaboratives have emerged from culinary schools, such as the CIA, to help accelerate progress towards healthier and more sustainable business practices across the food industry^(54)^. With the shift towards more home cooking in response to restaurant closures in the COVID-19 pandemic^(55)^, chefs have extended their influence beyond the restaurant into people’s homes through live virtual cooking demos, social media and meal kits. These growing platforms represent additional opportunities for chefs to educate the public and promote healthier and more sustainable eating behaviours.

The results from this study indicate that engagement with culinary students to advance public health goals may prove fruitful. An important area for further research is how to translate strong support for nutrition and environmental sustainability among culinary students into adoption of practices that advance healthy diets and sustainable food systems in their careers as chefs. Given the urgency of addressing diet-related health and environmental concerns, bold action is needed to close the gap between attitudes and action as a new generation of culinary professionals enters the workforce and become leaders in the food industry. Culinary schools can help address this gap by more seamlessly integrating nutrition and food systems training into their degree requirements and offering more courses, job shadowing and internship opportunities focussed on promoting healthy and sustainable foods. The food service industry is changing rapidly in response to growing consumer demand for plant-forward, health-centred and sustainably sourced foods, and culinary schools must adapt their programming accordingly. This shift will require more than one-off electives or a small number of required credits in nutrition and sustainability topics. The opportunity areas suggested by participants in this study form a preliminary framework for developing enhanced curricular and co-curricular strategies that teach and motivate culinary students to improve nutrition and sustainability when they become chefs, through their menu and purchasing decisions, their role as educators and influencers in the food system and their ability to make healthy and sustainable options more appealing and accessible to the public. These strategies must emphasise the substantial overlap between healthier and more sustainable diets, prioritising culinary and business practices that optimise benefits to human and environmental health. Culinary schools should seize the opportunity to empower their students with the necessary knowledge and skills to promote healthier and more environmentally conscious food choices in their careers as chefs.

### Limitations

A limitation of this study is its generalisability to other populations of culinary students since participants were recruited from a single culinary school and participation was voluntary, contributing to potential selection bias. While we did not have access to admissions data from other culinary schools for comparison, it should be noted that the CIA is considered as one of the more prestigious and expensive culinary schools in the USA^(56)^, with almost half of its students pursuing 4-year bachelors degrees in a number of specialised programs^(57)^. This may cater to a different demographic of students than other culinary schools and if so, our results would not necessarily reflect the attitudes, beliefs or career aspirations of all culinary students.

Additionally, our sample may not have been representative of the total student population at the CIA, with a higher proportion of female students completing the survey. The study sample had a greater proportion of Non-Hispanic White respondents and a smaller proportion of those identifying as Non-Hispanic Black, Hispanic and other race/ethnicity compared to the total student population. More than half of the participating students were in the first year of their program, which may have under-represented attitudes and beliefs of more experienced students as they approach graduation and prepare to enter the workforce. Also, survey questions were not taken from validated instruments or otherwise validated before use in this survey. Future research should focus on exploring nutrition and sustainability attitudes and beliefs among students at a broader sample of culinary schools and with working chefs and culinary professionals in various sectors of the food industry.

## Conclusion

This study supports the inclusion of chefs as valuable stakeholders in developing solutions to confront pressing diet and climate challenges. Culinary students represent the next generation of food industry leaders, and the majority of those participating in this study believe nutrition and sustainability are important considerations that chefs have a responsibility to address. There are differences in culinary students’ personal food choice priorities, attitudes and beliefs, especially across race/ethnicity, so efforts to engage culinary students should consider these disparities and aim to reconcile leading food choice priorities (e.g. taste, price, convenience) with health and sustainability values. Opportunities exist for public health practitioners and culinary educators to leverage culinary students’ support for nutrition and sustainable food systems into strategies that educate and inspire them to improve diet quality and environmental impact of the food system in their careers as chefs.

## Supplementary material

For supplementary material accompanying this paper visit https://doi.org/10.1017/S1368980021003578. click here to view supplementary material
