# Supplementary figures and images for: Attitudes and beliefs about how chefs can promote nutrition and sustainable food systems among students at a US culinary school
Source: Public Health Nutr. 2021 Aug 20;25(2):498–510. doi: 10.1017/S1368980021003578 (PMC8858328; doi:10.1017/S1368980021003578)

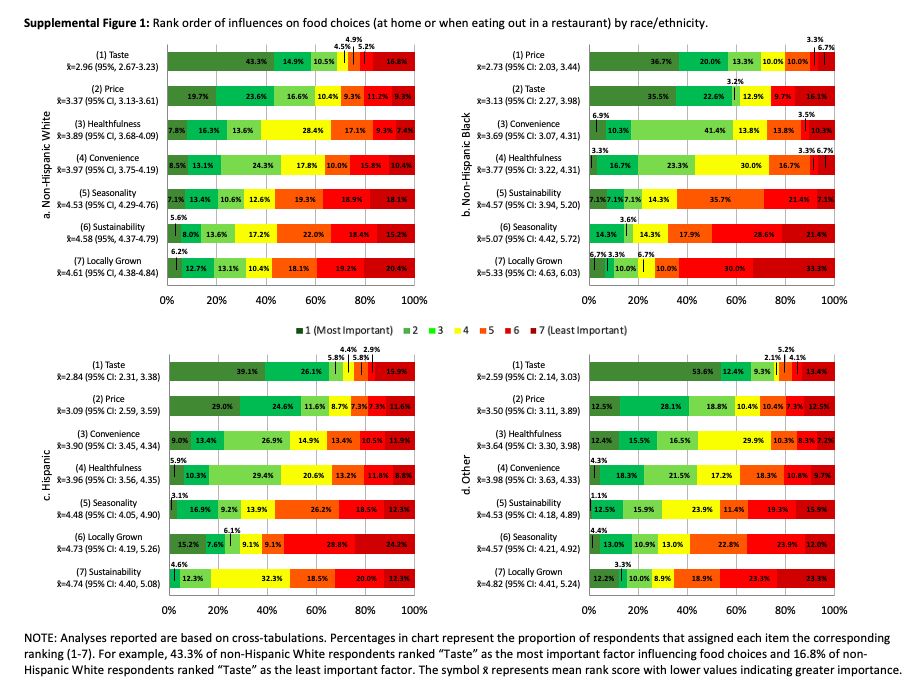

Supplement: Supplementary file 1 [file S1368980021003578sup001.png]
