# Supplementary material for: Attitudes and beliefs about how chefs can promote nutrition and sustainable food systems among students at a US culinary school
Source: Public Health Nutr. Author manuscript; Available in PMC 2023 Feb 20. (PMC8858328; doi:10.1017/S1368980021003578)
Supplement: Supplementary file 1 [file NIHMS1754967-supplement-Supplemental_FIgure_1.docx]

**Supplemental Figure 1:** Rank order of influences on food choices (at home or when eating out in a restaurant) by race/ethnicity.

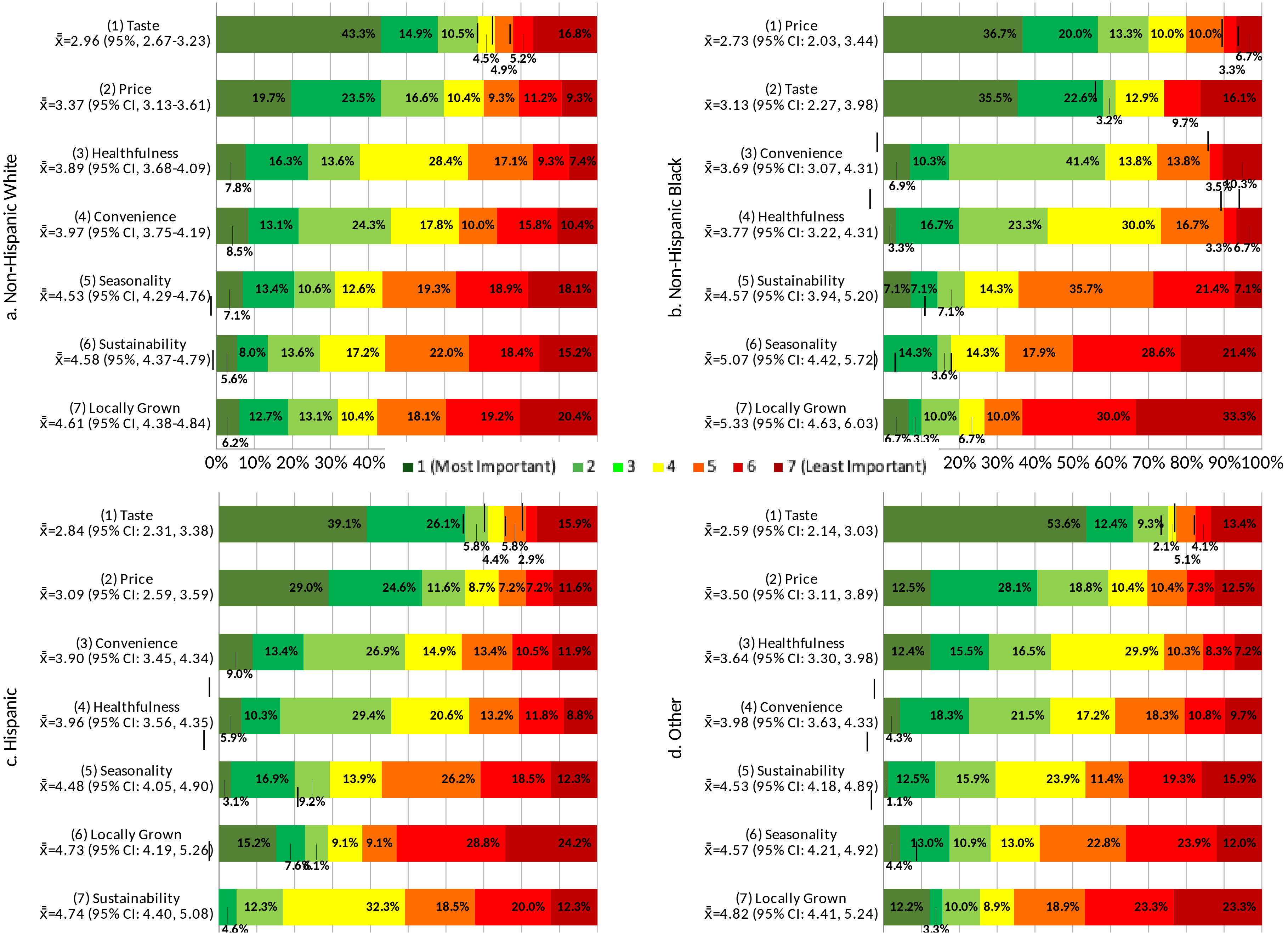


NOTE: Analyses reported are based on cross-tabulations. Percentages in chart represent the proportion of respondents that assigned each item the corresponding ranking (1-7). For example, 43.3% of non-Hispanic White respondents ranked "Taste" as the most important factor influencing food choices and 16.8% of non-Hispanic White respondents ranked "Taste" as the least important factor. The symbol x̄ represents mean rank score with lower values indicating greater importance.
